# GCN5L1-mediated TFAM acetylation at K76 participates in mitochondrial biogenesis in acute kidney injury

**DOI:** 10.1186/s12967-022-03782-0

**Published:** 2022-12-06

**Authors:** Tingting Lv, Yu Zhang, XingZhao Ji, Shengnan Sun, Li Xu, Weixia Ma, Yi Liu, Qiang Wan

**Affiliations:** 1 Center of Cell Metabolism and Disease, Jinan Central Hospital, Shandong University, Jinan, 250012 Shandong China; 2 Department of Cancer Center, Shandong Provincial Hospital Affiliated to Shandong First Medical University, Jinan, 250021 Shandong China; 3 Department of Allergy, Department of Pulmonary and Critical Care Medicine, Shandong Provincial Hospital Affiliated to Shandong First Medical University, Jinan, 250021 Shandong China; 4 Shandong Key Laboratory of Infections Respiratory Disease, Medical Science and Technology Innovation Center, Shandong First Medical University & Shandong Academy of Medical Sciences, Jinan, Shandong China; 5 Department of Allergy, Department of Pulmonary and Critical Care Medicine, Shandong Provincial Hospital, Shandong University, Jinan, 250021 Shandong China

**Keywords:** GCN5L1, Acetylation, TFAM, Mitochondrial biogenesis, Acute kidney injury

## Abstract

**Background:**

Mitochondrial dysfunction is an important pathogenic event in acute kidney injury (AKI). GCN5L1 is a specific acetyltransferase in mitochondria, which regulates glucose and fatty acid metabolism. However, the role of GCN5L1 in mitochondrial dysfunction and the pathogenesis of ischemic AKI are not fully understood.

**Methods:**

The protein level of GCN5L1 was detected by western blot assay. Acetylated proteomics was used to explore the level of acetylated TFAM. Duolink proximity ligation assay and co-immunoprecipitation were used to detect the interaction of TFAM and translocase of outer membrane 70 (TOM70). mtDNA copy number, the expression of mitochondrial electron transport chain complexes, the number and morphology of mitochondria were measured. The renal injury of AKI mice was reflected by the levels of creatinine and urea nitrogen and the pathological changes of renal tissue.

**Results:**

We showed that GCN5L1 was highly expressed in vivo and in vitro and renal tubules specific knockdown of GCN5L1 could effectively attenuate AKI-induced mitochondrial impairment. Besides, acetylated proteomics revealed that acetylated TFAM was significantly upregulated in AKI mice kidney, which reminded us that TFAM might be an acetylating substrate of GCN5L1. Mechanistically, we evidenced that GCN5L1 could acetylate TFAM at its K76 site and subsequently inhibited its binding to TOM70, thereby reducing TFAM import into mitochondria and mitochondrial biogenesis. Clinically, GCN5L1 and acetylated TFAM were positively correlated with disease severity (all p < 0.05).

**Conclusions:**

In sum, these data demonstrated an unrecognized regulating mechanism of GCN5L1 on TFAM acetylation and its intracellular trafficking, and a potential intervening target for AKI associated mitochondrial disorders as well.

**Supplementary Information:**

The online version contains supplementary material available at 10.1186/s12967-022-03782-0.

## Introduction

Acute kidney injury (AKI) is an important clinical syndrome with increased short- and long-term morbidity and mortality, which is often caused by ischemia–reperfusion (IR), nephrotoxic drugs, sepsis and urinary tract obstruction [1]. At present, effective therapies for AKI are limited, partly due to incompletely understand of the pathogenesis and molecular mechanism of renal damage. Therefore, it is urgent to find new targets for the intervention to inhibit its progression.

The proximal renal tubules are rich in mitochondria which generate sufficient quantities of ATP through the process of oxidative phosphorylation (OXPHOS) to maintain mitochondrial homeostasis, which is essential for normal kidney function [2]. Mitochondria are the major intracellular sources which are extremely vulnerable to damage during different cellular energetic conditions. Mounting evidences have demonstrated that mitochondrial function is vital for AKI and mitochondrial biogenesis plays a beneficial role in kidney injury and repair the function of kidney after AKI [3, 4]. It has been reported that the amounts of mitochondria were greatly reduced in rat kidney after I/R accompanied with mitochondria structural changes [5]. Besides, several clinical trials show that the kidney cortical mtDNA copy number is decreased and urinary mitochondrial DNA (UmtDNA) levels which correlated with renal injury in sepsis-induced AKI patients are considered to be a valuable biomarker for the occurrence of AKI [6]. As an important regulation mode of mitochondrial homeostasis, the decline of mitochondrial biogenesis inevitably leads to the reduction of mitochondrial quantity and inhibition of mitochondrial respiration, which may further induce mitochondrial damage and lead to renal intrinsic cell death, kidney injury and possible organ failure [7]. Therefore, seeking intervening measures for promoting mitochondrial biogenesis is a significant way to prevent AKI.

Mitochondrial transcription factor A (TFAM) is the most abundant mtDNA packaging protein that is transported from the cell nucleus to mitochondria and is required for mtDNA maintenance, transcription, and replication [8]. Whole deletion of TFAM is embryonically lethal, but tissue-specific lack of TFAM disrupts respiratory chain function and generates a variety of alterations that recapitulate important phenotypes of human mitochondrial diseases [9, 10]. Several of posttranslational modifications of TFAM have been reported. Ubiquitination and phosphorylation of TFAM impaired the transcriptional activity [11, 12]. Lysine acetylation of TFAM is also shown by Graeme A King and colleagues [13]. However, which is responsible for the acetylation of TFAM is unclear.

General control of amino acid synthesis 5 like-1 (GCN5L1), a recently identified acetyltransferase which sequence homology to the nuclear acetyltransferase GCN5. GCN5L1 first aroused interest in understanding its role in lysosomes biogenesis and endosome–lysosome trafficking in the cytoplasm [14]. Subsequent studies have explored that GCN5L1 could also be located in mitochondria and modulate mitochondrial protein acetylation to regulate multiple mitochondrial biological functions. Moreover, GCN5L1 could also be used as a cell energy sensor to mediate a variety of cell biological reactions including mitochondrial metabolism by responding to different energy states of cells [15]. Our previous study found that mitochondrial GCN5L1 drives mitochondrial ROS and fatty acid oxidation [16, 17]. Herein, we further explore the critical role of GCN5L1 in mitochondrial biogenesis in AKI. We first reported that GCN5L1 was significantly elevated both in vivo in human and mice AKI kidney and in vitro renal tubular epithelial cells (TECs) treated with hypoxia/reoxygenation, while reducing GCN5L1 expression could effectively attenuate kidney damage in AKI. Furthermore, GCN5L1 could acetylate TFAM at its K76 site, and then impair its binding with the translocase of outer mitochondrial membrane 70 (TOMM70), which affected the import of TFAM into mitochondrial and inhibited its DNA-binding capabilities, thereby impairing mitochondrial biogenesis. These results suggested that GCN5L1-mediated TFAM acetylation might be a key regulator and energetic sensor of mitochondrial biogenesis.

## Methods

### Human subjects

Tissue sections were obtained from patients with acute kidney injury confirmed by renal biopsy from department of Pathology, Cheeloo College of Medicine, Shandong University. Control samples were collected from normal kidney tissues of patients undergoing nephrectomy of renal carcinoma without other renal diseases. All procedures were approved by the institutional review committee of Cheeloo College of Medicine, Shandong University (ECSBMSSDU2018-1-045) after written informed consent obtained from the patients.

### Animal experiments

C57BL/6J male mice (6–8 weeks old, 6 mice in each group) were pursued from Shandong University Experimental Animal Centre and were housed under standard conditions and cared for according to the institutional guidelines for animal care. All animal experiments were approved by Institutional Animal Care and Ethics Committee of Shandong University (No. S077).

### Intrarenal adeno-associated virus delivery

AAV2/9-HU6-shGCN5L1 (titer: 1 × 10^12^ vg/ml) and AAV2/9-HU6-Scramble (titer: 1 × 10^12^ vg/ml) were purchased from Genomeditech (Shanghai, China). The target sequence of shGCN5L1 was 5′-GAAGAGGAGGAGAGAAGCTAT-3′. The sequence of negative control was 5′-TTCTCCGAACGTGTCACGT-3′. Mice were anesthetized by intraperitoneal injection of 1% pentobarbital sodium (35–40 mg/kg), then the kidneys were exposed via a back incision. After diluted in 30 μl sterile 0.9% sodium chloride solution, AAV9-HU6-shGCN5L1 or AAV9-HU6-Scramble (100 μl/kidney) was injected into the kidney parenchyma in four or five sites using a 30G needle.

### Mice models

Mice were treated with renal ischemia 1 month after AAV injection. 1% pentobarbital sodium (35–40 mg/kg) was used to intraperitoneal anesthesia, and mice were then put in a prone position to fix on a 37 °C heating plate. Acute kidney injury was induced by bilateral renal pedicles clamping for 30 min. Sham mice underwent surgery without renal pedicle clamp. Mice were sacrificed at 48 h after reperfusion, and the kidney tissues, blood, and urine were collected for further analysis.

### Renal function measurement

Blood samples were centrifuged at 3000 rpm for 5 min at 4 °C to collect serum. Serum creatinine and urea nitrogen were measured by commercial kits based on manufacturer’s instructions respectively (Nanjing Jiancheng Bioengineering Institute, C011-2-1, C013-2-1).

### Human renal tubular epithelial cell line

Human renal proximal tubular epithelial cells (TECs) were purchased from the American Type Culture Collection (ATCC, Manassas, VA) and cultured in low glucose Dulbecco’s Modified Eagle Medium (DMEM) supplemented with 10% FBS and 1% penicillin–streptomycin at 37 °C in a 5% CO_2_ incubator. For the H/R model, TECs were firstly cultured in medium without nutrients (glucose-free, serum-free) for 12 h in a humidified hypoxic incubator with 1% O2, 5% CO_2_ and 94% N2. After hypoxic treatment, cells were transferred into a normal incubator (5% CO_2_ and 95% air) with regular culture medium and cultured for 12 h [18]. Control cells were incubated in complete culture medium in a regular incubator.

### Transfection

Cells were plated at a density of 6 × 10^4^ cells/well in a 6-well plate. After 12–24 h, cells were transfected with specific siRNAs targeting GCN5L1 or TFAM according to the manufacturer’s protocol of RiboBio (Guangzhou, CN). Lipofectamine 3000 (Invitrogen) was used to transfect the GCN5L1 or TFAM overexpression plasmid when the cells reached 70–80% confluence. pDsRed2-Mito was applied for red fluorescence labeling of mitochondria which could then be transfected into cells using Lipofectamine 3000 (Invitrogen), as described previously [17].

### Western blot analyses

Proteins from lysed cells were fractionated by 10% or 12% SDS-PAGE and transferred to 0.45 μm PVDF nitrocellulose membrane. Then nonspecific binding sites were blocked with 5% skim milk in TBST for 2 h at room temperature (Millipore, USA). The membranes were incubated with primary antibodies overnight at 4 °C (Antibodies were described in Additional file 1: Table S1). The next day, all membranes were washed three times with TBST and incubated with an HRP-conjugated secondary antibody. Finally, proteins bands were visualized by Western Chemiluminescence (Millipore, USA) on a Storm 860 imager (Molecular Devices).

### Development of anti-TFAM (acetyl K76) antibody

The polyclonal antibody specific for the acetylated TFAM at K76 (anti-acetylated K76-TFAM) was produced in Chinapeptides company. Rabbits were immunized with the acetylated human TFAM peptide (QNPDAK(Ac)TTELIRC) where aceK76 represents the acetylated K76. Antisera from the immunized rabbits were first depleted with the unacetylated peptide (QNPDAKTTELIRC) and then affinity-purified using the acetylated peptide.

### Quantitative real-time PCR

Total RNA was extracted using Trizol Reagent (Invitrogen). cDNA was reversed using PrimeScript™ RT reagent Kit (TAKARA) according to the manufacturer’s instructions. Quantitative real-time PCR was performed with the TB Green Premix Ex Taq™ II (TAKARA, No. RR820L) on SYBR Green I/HRM Dye PCR System (Roche 480II). Expression levels of the detected genes were normalized to β-actin expression levels (Primers are listed in Additional file 1: Table S2).

### Chromatin immunoprecipitation assay (ChIP)

Cells were cross-linked with 1% formaldehyde at 37 °C for 10 min. Glycine was added to stop the reaction. Then, cells were resuspended in lysis buffer containing protease inhibitors. The chromatin was disintegrated into small fragments by sonication and then incubated with antibody against TFAM and Protein A/G Magnetic beads at 4 °C overnight. The next day, magnetic beads were separated using magnetic frame. Immunoprecipitated DNA was retrieved from the beads with elution buffer. Expression of the mitochondrial DNA was quantified by qRT-PCR using specific primers (F-AAGAACCCTAACACCAGCCTAAC; R-AAGAACCCTAACACCAGCCTAAC’). Rabbit IgG was used as the negative antibody control.

### Immunoprecipitation and co-immunoprecipitation

The immunoprecipitation and co-Immunoprecipitation were performed using Crosslink IP kit (Thermo Scientific Pierce, 26147) and Co-IP kit (Thermo Scientific Pierce, 26149) according to the manufacturer’s instructions respectively. For the immunoprecipitation assay, the cells were lysed with lysis buffer and cell lysates were incubated with 10 μg acetylation antibody and cross-linked resin on a rotator overnight at 4 °C with mouse-IgG antibody as a negative control. The next day, protein complexes bound to the antibody were eluted by elution buffer and subjected to western blot assay using mouse TFAM antibody. For the co-immunoprecipitation assay, 10 μg TFAM antibody was immobilized with Amino Link Plus coupling resin for 2 h and then incubated with cell lysates at 4 °C overnight. After incubation, the resin was washed with elution buffer and eluted proteins were analyzed by western blot assay using rabbit HSP70 antibody, rabbit TOM70 antibody rabbit, TOM40 antibody, rabbit TOM20 antibody, rabbit TIMM44 antibody and rabbit TIMM17A antibody.

### Histopathology and immunohistochemistry

Kidney tissues were fixed in 4% paraformaldehyde, embedded in paraffin, and sectioned at 5 μm intervals. After being deparaffinized and rehydrated, selected sections for H/E staining were stained with hematoxylin and eosin. For immunohistochemistry, sections were placed in sodium citrate buffer to retrieval antigens. Then slides were incubated with the primary antibodies diluted in PBS at 4 °C overnight. After three 5 min washes with PBS, sections were incubated with HRP-conjugated secondary antibody at 37 °C for 1 h and then visualized with diaminobenzidine, counterstained with hematoxylin, dehydrated, and sealed with neutral gum. Finally, images were obtained under a Nikon microscope imaging system (Nikon, Tokyo, Japan).

### Immunofluorescence staining

After washing with PBS, the cells were fixed in 4% paraformaldehyde for 15 min at room temperature and permeabilized with 0.1% Triton X-100 diluent in PBS for another 15 min at room temperature. Then, 1% goat serum (Solarbio, SL038) was used to block nonspecific sites for 1 h at room temperature followed by overnight incubation with primary antibodies diluted in 1% goat serum at 4 °C. Antibody staining was visualized with Alexa 594 goat anti-rabbit or Alexa Fluor 488 goat anti-mouse. Finally, DAPI (Solarbio, C0060) was added to stain the cell nuclei. Images were obtained under a Nikon microscope (Nikon, Tokyo, Japan) or LSM 700 Laser scanning confocal microscope (Zeiss, Germany).

### Mitochondrial DNA quantification

Total DNA was harvested from treated cells or mice kidneys using FastPure Cell/Tissue DNA Isolation Mini Kit (Vazyme, DC102-01). The mtDNA copy number was expressed as the mitochondrial DNA (mtDNA) /nuclear DNA (nDNA) ratio. Mitochondrial gene D-Loop 2 (mtDNA), mitochondrial gene cytochrome c oxidase I (mtDNA), nuclear gene G6PC (nDNA) and nuclear gene β-actin (nDNA) were determined by quantitative real-time PCR-based method as described previously [17]. Primers are listed in Additional file 1: Table S2.

### Transmission electron microscopy

Treated cells or mice kidney were gently collected and fixed with ice-cold 2.5% glutaraldehyde and 1% osmium tetroxide. After being dehydrated and embedded, samples were sliced into ultrathin sections, which were then evaluated under JEM-100sX electron microscopy to observe mitochondrial abundance and morphology. The number of mitochondria was estimated per square micron of the field was estimated using Image-Pro plus 6.0.

### Protein-binding site prediction

The experimental structure of TFAM was directly obtained from RCSB PDB database (http://www.rcsb.org/) with the accession of 3TMM. The protein structure of GCN5L1, which was predicted by AlphaFold, a highly accurate artificial intelligence-based computational structure modeling method, was retrieved from the UniProt database (http://www.uniprot.org/). Then the structures of these proteins were submitted to the PRISM tool (http://cosbi.ku.edu.tr/prism) to predict their potential interaction interface. Finally, the prediction results were visualized by the PyMol tool (http://pymol.org).

### Measurement of bioenergetic profile

The treated cells were plated in XF96 cell culture microplates (Seahorse Bioscience). XF96 extracellular flux analyzer (Seahorse Biosciences, North Billerica, MA, USA) were employed to measure the oxygen consumption rate of cells. After measurement of basal OCR, cells were treated with oligomycin (2 μM), FCCP (1 μM), and rotenone/antimycin A (5 μM) to generate a bioenergetic profile, as described previously [19].

### Measurement of oxygen consumption

Mitochondrial oxygen consumption of mice renal cortex was measured by Clark-type oxygen electrode (Hansatech Instruments, UK). Mitochondria were isolated from freshly kidney tissues and suspended in respiration buffer to obtain a final concentration of 0.5 mg/ml. The respiratory substrates and inhibitors were added to detect oxygen consumption of the respiratory chain complexes including Complex I (5 mM Glutamate plus 5 mM Malate, 2 mM Rotenone), Complex II + III (5 mM Succinate, 0.1 mM Antimycin A) and Complex IV (1.2 mM TMPD, 6 mM KCN).

### Proximity ligation assay

Cells were grown on glass slides in 24-well plates. Then coverslips were fixed in 4% paraformaldehyde and permeabilized with 0.1% Triton X-100. The interaction of TFAM with acetyl-lysine, HSP70, TOM70, TOM40, TOM20, TIMM44 and TIMM17A was detected by using Duolink™ In Situ proximity ligation assay kit (Duolink, Sigma) to detected as described by the manufacturer. Green fluorescence reactions in cells were captured by Nikon microscope (Nikon, Tokyo, Japan) or TCS SP8 Laser scanning confocal microscope (Leica, Germany) and analyzed by Image J.

### Statistical analysis

All the statistical analyses were performed using GraphPad Prism 7 software. Two-tailed unpaired Student’s t-test was used to detect the difference between the two groups. Multiple groups were compared using one-way ANOVA test. P < 0.05 was considered to be significant.

## Results

### The expression of GCN5L1 is significantly upregulated in AKI and specific tubular GCN5L1 knockdown alleviates I/R-induced kidney damage

**Fig. 1:**
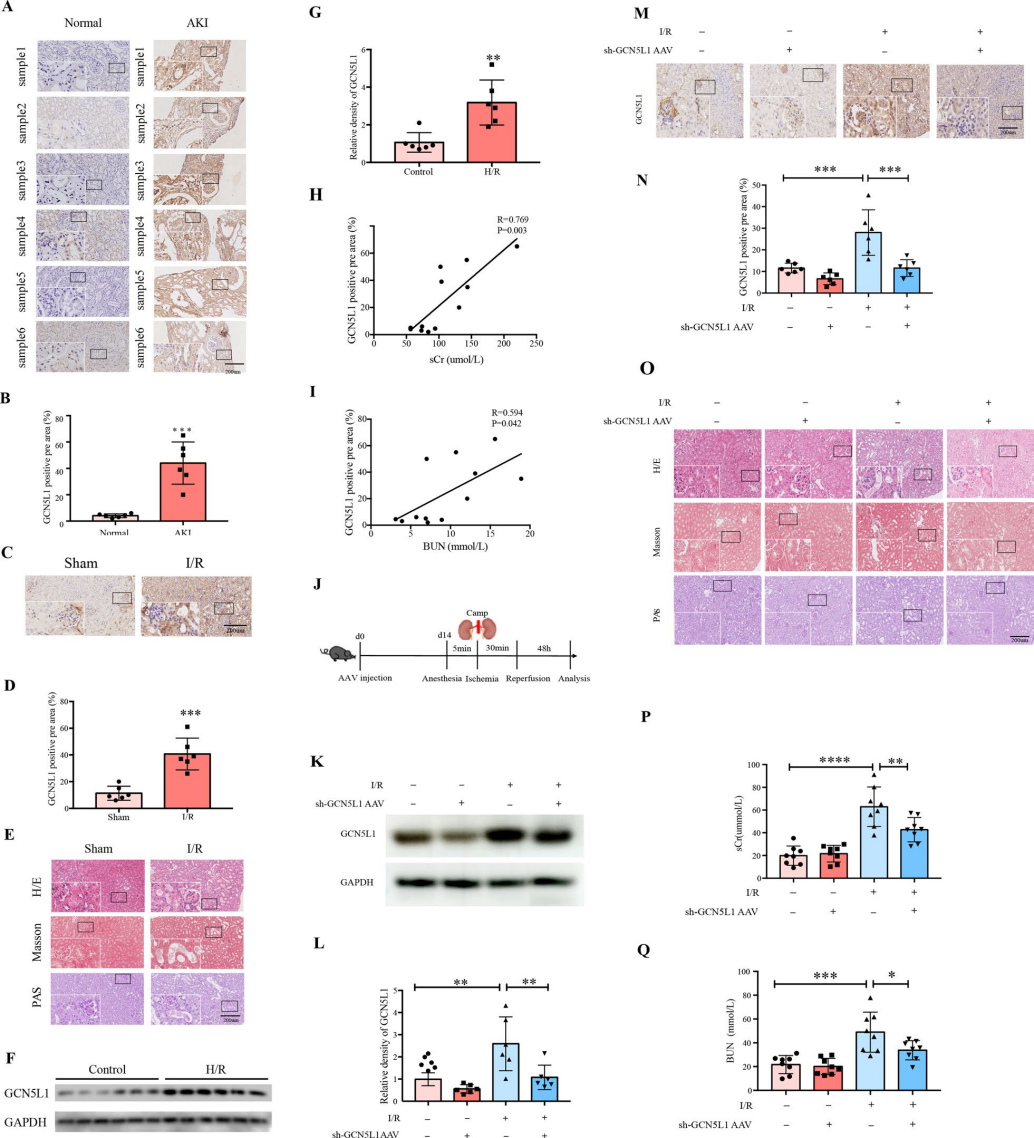
The expression of GCN5L1 is significantly upregulated in AKI and specific tubular GCN5L1 knockdown alleviates I/R-induced kidney damage. **A**, **B** Immunohistochemistry staining for GCN5L1 expression in kidney sections. **C**, **D** Immunohistochemistry staining for GCN5L1 expression in kidney sections of AKI mice model. **E** Representative photomicrographs of H/E, MASSON and PAS staining in kidney sections of AKI mice models. **F**, **G** Western blot analysis of GCN5L1 protein expression in hypoxia-reoxygenation induced TECs. **H** Positive correlation (Spearman r = 0.769, P = 0.003) between GCN5L1 IHC staining levels and serum creatinine in all subjects. **I** Positive correlation (Spearman r = 0.594, P = 0.042) between GCN5L1 IHC staining levels and BUN in all subjects. **J** Experimental design of GCN5L1 knockdown mice with acute kidney injury. **K**, **L** Western blot analysis for the expression of GCN5L1 in the kidneys from GCN5L1 knockdown mice with acute kidney injury. **M**, **N** Immunohistochemistry staining for GCN5L1 expression in kidney sections of mice. **O** Representative photomicrographs of H/E, MASSON and PAS staining in kidney sections of mice. **P**, **Q** Serum creatinine and BUN levels of GCN5L1 knockdown mice with acute kidney injury. *P < 0.05, **P < 0.01, and ***P < 0.001

First, we investigated the expression of GCN5L1 in kidney samples from both ischemic kidney injury patients and bilateral renal pedicle clamping-induced AKI mice. Immunohistochemistry (IHC) staining confirmed the augmented expression of GCN5L1 in renal tissues from acute kidney injury patients (Fig. 1A, B), and renal ischemia/reperfusion mice (Fig. 1C, D). Renal morphological examination by H/E staining, Masson’s trichrome staining, and PAS staining indicated that renal damage was aggravated in I/R-induced mice kidney compared with the sham operation group (Fig. 1E). Notably, quantification of IHC staining demonstrated that the level of GCN5L1 was positively correlated with the serum creatinine (Spearman r = 0.769, P = 0.003, n = 6) (Fig. 1H) and blood urea nitrogen (Spearman r = 0.594, P = 0.042, n = 6) (Fig. 1I) in AKI patients. Consistent with the results in vivo, hypoxia/reoxygenation induced protein levels of GCN5L1 in TECs also showed the same tendency (Fig. 1F, G). Thus, these data suggested that GCN5L1 might be involved in the pathogenesis of ischemic kidney injury.

We further demonstrated the role of GCN5L1 in in the pathogenesis of AKI. First, we generated kidney specific GCN5L1 knockdown mice by kidney cortex local injection of sh-GCN5L1 AAV and then bilateral renal pedicle clamping-induced AKI to observe whether reducing GCN5L1 could have protective effects (Fig. 1J). The efficiency of GCN5L1 knockdown in the kidney was confirmed by western blotting (Fig. 1K, L), and IHC staining (Fig. 1M, N). Kidney morphological detection demonstrated that I/R induced tubular epithelial cell necrosis, brush border disappearance and tubular dilation were ameliorated by GCN5L1 knockdown (Fig. 1O). Blood biochemistry analysis also revealed that additional GCN5L1 knockdown effectively decreased blood urea nitrogen (BUN) and creatinine (sCr) levels (Fig. 1P, Q). Hence, these data suggested that reducing GCN5L1 expression was an effective intervening target in AKI.

### GCN5L1 acetylated TFAM at K76 site

**Fig. 2:**
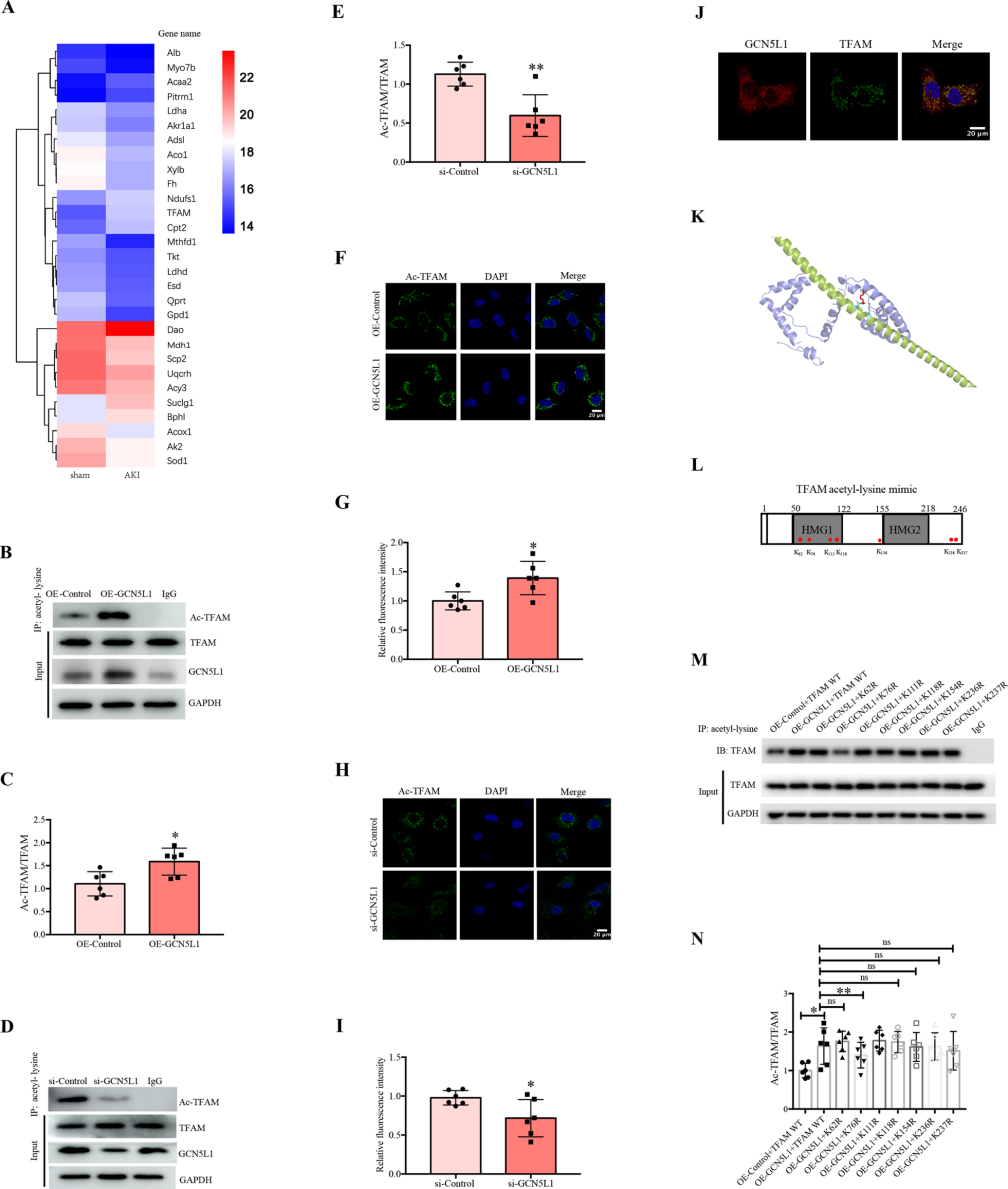
GCN5L1 acetylated TFAM at K76 site. **A** The heatmap of mass spectrometry-based acetylated proteomics. **B–E** Acetylation level of TFAM was detected by immunoprecipitation in GCN5L1 overexpression or knockdown TECs. **F–I** Acetylation level of TFAM was detected in GCN5L1 overexpression or knockdown TECs visualized by Duolink proximity ligation assay. **J** The co-localization of TFAM with GCN5L1 was detected by immunofluorescent staining. **K** Diagram for TFAM potential acetylation sites and the construction of mutants. **L**, **M** Acetylation level of TFAM was detected by immunoprecipitation in TECs co-transfected with GCN5L1 and TFAM site mutation overexpressing plasmids. **N** The structure model of GCN5L1 interaction with TFAM. ns: not significant, *P < 0.05, and **P < 0.01

Considering GCN5L1 is an acetyltransferase and is known to acetylate several mitochondrial proteins such as those involved in glucose and fatty acid metabolism, we used acetylated proteomics to find the potential acetylating substrate of GCN5L1. The mass spectrometry-based acetylated proteomics identified 29 differentially expressed acetylated proteins in AKI mice kidney, among these proteins TFAM raised our interests as it’s a key modulator of mitochondrial biogenesis (Fig. 2A). Then we performed the following experiments to explore the relationship between GCN5L1 and TFAM. First, we demonstrated that GCN5L1 does affect acetylated TFAM levels. As Fig. 2B–E showed, GCN5L1 overexpression induced a significant elevation of acetylated TFAM, while GCN5L1 knockdown diminished it to very low levels. Such the effects were also confirmed by the PLA assay (Fig. 2F–I). Second, we performed immunostaining study for GCN5L1 and TFAM in TECs and found these two molecular co-localized, providing further evidences supporting that GCN5L1 could acetylate TFAM directly (Fig. 2J). Subsequently, we determined the potential acetylating site of TFAM molecular as followed (Fig. 2K). With bioinformatics analysis (BIOCUCKOO analysis, http://pail.biocuckoo.org/wsresult.php) and literature reviewing, a total of 7 candidate sites were screened and analysed accordingly, including K62, K76, K111, K118, K154, K236 and K237^11,17^. After TFAM mutants with each lysine residue above replaced by arginine were constructed respectively (Fig. 2L), these TFAM variants were co-transfected with GCN5L1-overexpressing plasmids, and then the acetylation level of TFAM was analyzed by immunoprecipitation (IP). Results are shown in Fig. 2M and N. The acetylation of TFAM was increased by GCN5L1 overexpression, which was significantly reduced by TFAM K76 mutant, but not by other sites variants, indicating that GCN5L1 selectively acetylated TFAM at its K76 site. Finally, based on the structure of TFAM, we predicted that the two models of GCN5L1 would interact with TFAM using a software. The acetylated lysine LYS76 and its interacting residue on GCN5L1 (GLU64) are highlighted in red and cyan, respectively (Fig. 2K).

### GCN5L1 affects the intracellular distribution of TFAM and then alters TFAM-mtDNA binding capability

**Fig. 3:**
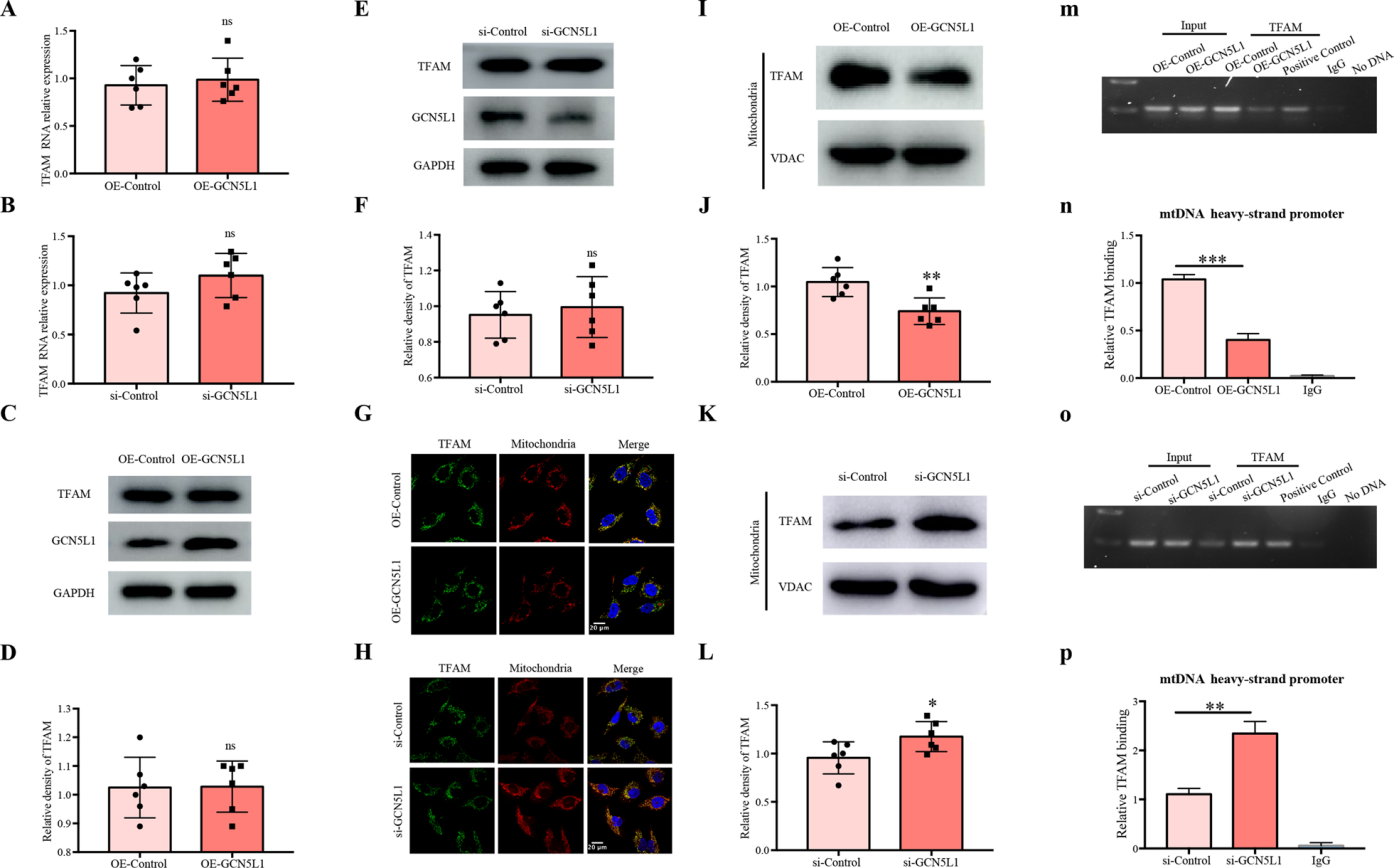
GCN5L1 affects the intracellular distribution of TFAM and then alters TFAM-mtDNA binding capability. **A**, **B** mRNA levels of TFAM were detected by qRT-PCR in GCN5L1 overexpression or knockdown TECs. **C–F** Protein levels of TFAM were detected by western blot in GCN5L1 overexpression or knockdown TECs. **G**, **H** Confocal microscopy for the accumulation of TFAM in mitochondria in TECs co-transfected GCN5L1 overexpressing plasmid or GCN5L1 silencing siRNA with pDsRed2-Mito plasmid. **I–L**Western blot showed the TFAM expression in isolated mitochondrial protein in GCN5L1 overexpression or knockdown TECs. **M–P** The binding of TFAM with mtDNA revealed by ChIP assay in GCN5L1 overexpression or knockdown TECs and rabbit IgG was used as a negative control. ns: not significant, *P < 0.05, **P < 0.01, and ***P < 0.001

We further asked how GCN5L1 induced TFAM K76 acetylation affected the function of TFAM. The study was performed via the following steps. First, GCN5L1 did not affect TFAM expression, as neither total mRNA nor protein contents of TFAM was altered by GCN5L1 overexpression or knockdown (Fig. 3A–F). Second, GCN5L1 obviously affected the intracellular distribution of TFAM. As shown in Fig. 3G–H, confocal microscope analysis revealed a significant elevation of cytoplasmic TFAM contents and a simultaneous reduction of mitochondrial TFAM contents by GCN5L1 overexpression, while GCN5L1 knockdown showed opposite effects. Our following western blot studies also demonstrated a corresponding decrease or increase of mitochondrial TFAM contents by GCN5L1 overexpression or knockdown respectively (Fig. 3I–L). Third, the modulation of TFAM intracellular distribution by GCN5L1 was accompanied with altered TFAM-mtDNA binding capabilities, which were investigated using ChIP assay. As Fig. 3M–P showed, GCN5L1 overexpression led to an approximately 0.4fold decrease of TFAM-mtDNA binding, while GCN5L1 knockdown elevated it about 2.5fold. Collectively, these data suggested that, although GCN5L1 did not affect TFAM expression, its mediated acetylation of TFAM K76 site may modulate the translocation of TFAM to mitochondria and then affect the transcriptional activity of TFAM.

### GCN5L1-induced TFAM K76 acetylation inhibits the binding of TFAM to translocase TOM70

**Fig. 4:**
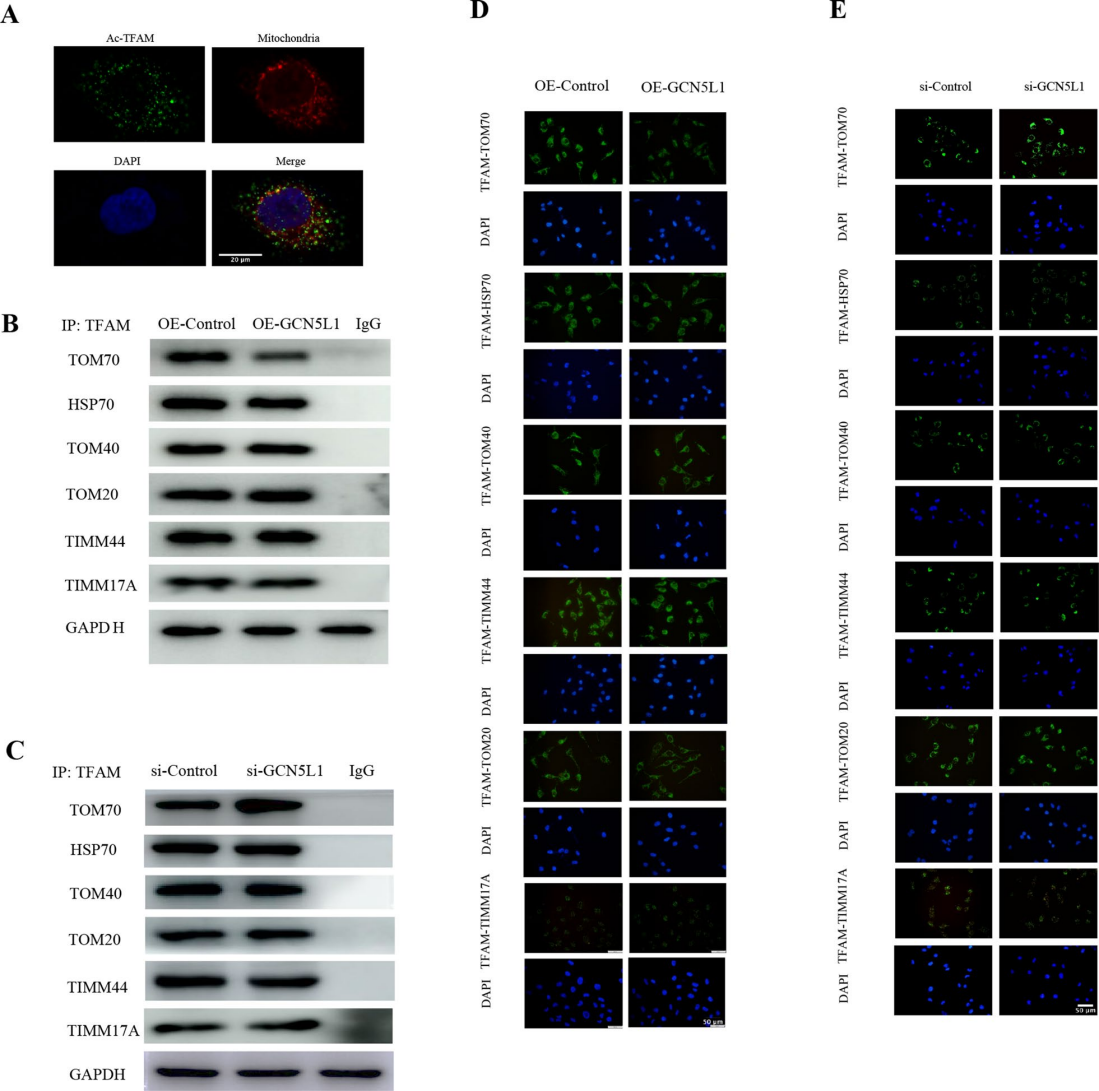
GCN5L1-induced TFAM K76 acetylation inhibits the binding of TFAM to translocase TOM70. **A** Confocal microscopy for the location of acetylated TFAM and mitochondria in TECs. **B** Immunoblotting of TFAM interacting with TOM70, HSP70, TOM70, TOM40, TOM20, TIMM44 and TIMM17A from GCN5L1 overexpression TECs was identified by Co-IP. **C** Immunoblotting of TFAM interacting with TOM70, HSP70, TOM70, TOM40, TOM20, TIMM44 and TIMM17A from GCN5L1 knockdown TECs was identified by Co-IP. **D** The binding of TFAM with mitochondrial membrane translocases and chaperone protein including TOM70, HSP70, TOM40, TOM20, TIMM44 and TIMM17A was shown by Duolink proximity ligation assay in GCN5L1 overexpression TECs. Scale bars = 50 μm. **E** The binding of TFAM with mitochondrial membrane translocases and chaperone protein including TOM70, HSP70, TOM40, TOM20, TIMM44 and TIMM17A was shown by Duolink proximity ligation assay in GCN5L1 knockdown TECs. Scale bars = 50 μm

GCN5L1 is known to localize at both cytoplasm and mitochondria, while TFAM is a nuclear-encoded protein and should be transported from cytoplasm into mitochondria, thus we first investigated whether GCN5L1-induced TFAM acetylation occurred at cytoplasm or within mitochondria. Using PLA assay (Fig. 4A), we demonstrated that most of GCN5L1-induced acetylated TFAM did not co-localize with mitochondria, suggesting GCN5L1 induced TFAM acetylation occurred in cytoplasm. Taking in account of the above data that GCN5L1 overexpression could led to the TFAM cytoplasm accumulation, it is reasonable to hypothesize that GCN5L1-induced TFAM acetylation might impair its translocation into mitochondria. To test this hypothesis, we employed bioinformatics analysis (STRING protein–protein interaction networks, https://cn.string-db.org/) and reviewed related literature, and screened a total of 6 potential mitochondrial transporting proteins for TFAM, including HSP70, TOM70, TOM40, TOM20, TIMM44 and TIMM17A [18]. Subsequently, we observed whether GCN5L1-induced TFAM acetylation could affect the binding between TFAM and these transporting proteins. The PLA assay showed that although all 6 transporters could bind to TFAM, only the binding of TFAM with TOM70 was obviously decreased by GCN5L1 overexpression, leaving other transporters unaffected including HSP70, TOM70, TOM40, TOM20, TIMM44 and TIMM17A (Fig. 4D). Such effects were also confirmed by co-immunoprecipitation assay (Fig. 4B). These results suggested that GCN5L1-induced TFAM K76 acetylation might selectively inhibit its binding to TOM70. Then, such conclusions were verified by GCN5L1 knockdown results. As Fig. 4C and E showed, GCN5L1 knockdown elevated the binding of TFAM to TOM70, without altering the binding of TFAM to other 5 transporters. Thereby, we demonstrated that GCN5L1 mediated TFAM K76 acetylation could selectively inhibit TFAM binding to TOM70. As TOM70 is a known translocase mainly responsible for facilitating cargoes entering across OMM^19^, GCN5L1 induced TFAM K76 acetylation and decreased binding capability to TOM70 could unavoidably lead to TFAM accumulation outside mitochondria.

### GCN5L1 inhibition alleviates I/R-induced mitochondrial biogenesis impairment in vivo and in vitro

**Fig. 5:**
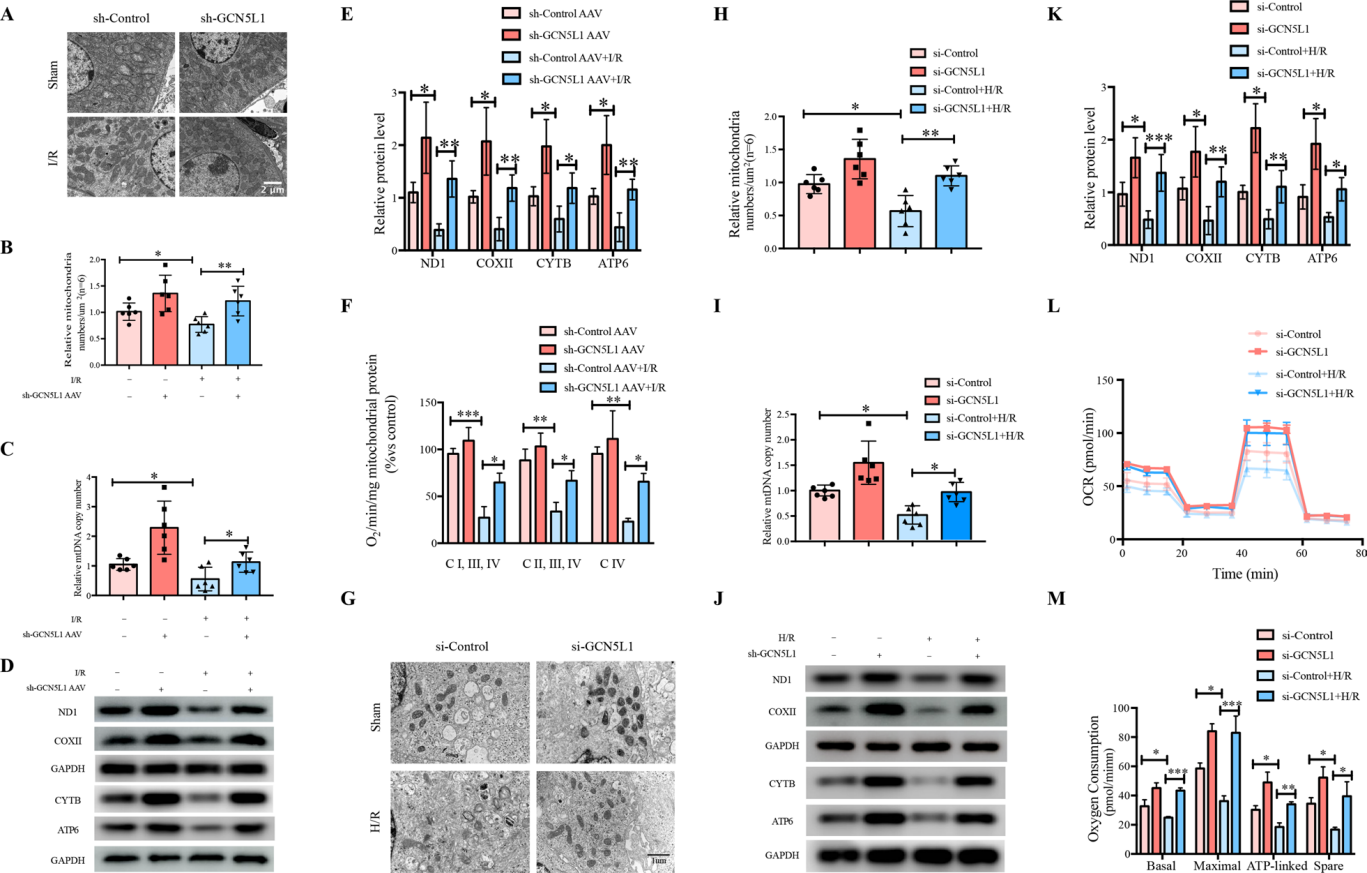
GCN5L1-induced TFAM K76 acetylation inhibits the binding of TFAM to translocase TOM70. **A**, **B** Electron microscopy analysis of kidney for mitochondrial number and morphology with semi-quantification of mitochondrial abundance. **C** mtDNA copy number in GCN5L1 knockdown mice kidney treated with I/R. **D**, **E** Protein levels of ND1, COXII, CYTB and ATP6 were detected by western blot in GCN5L1 knockdown mice kidney treated with I/R. **F** Oxygen consumption was recorded by clark-type electrode in GCN5L1-KD mice kidney treated with I/R. **G**, **H** Electron microscopy analysis of TECs for mitochondrial number and morphology with semi-quantification of mitochondrial abundance. **I** mtDNA copy number in H/R induced TECs. **J**, **K** Protein levels of ND1, COXII, CYTB and ATP6 were detected by western blot in hypoxia-reoxygenation induced TECs. **L**, **M** Mitochondrial OXPHOS were analyzed with basal respiration, maximal respiration, ATP production and spare respiratory capacity in GCN5L1 knockdown TECs under H/R treatment respectively. *P < 0.05, **P < 0.01, and ***P < 0.001

Tubular epithelial cells are one of the most highly energy-demanding cell types within body whose energy supply mainly derives from mitochondrial respiration [20]. Mitochondrial dysfunction is the main basic pathogenic event of AKI. As a specific transcriptional factor for mitochondrial DNA (mtDNA), TFAM was proposed as a crucial player in the process of mitochondrial biogenesis [21]. As the role of GCN5L1-mediated TFAM acetylation in the mitochondrial importing machinery of TFAM was established, we were interested in determining whether this mechanism could act as an energetic sensor of mitochondria when challenged by energy stresses such as serum deprivation. Electron microscopy analysis confirmed that I/R induced mitochondrial damage, such as reduced mitochondrial abundance, loss of cristae and presence of swollen mitochondria, were also ameliorated by GCN5L1 knockdown (Fig. 5A, B). Besides, GCN5L1 knockdown was effective in elevating kidney tissue mtDNA copy number and expression of OXPHOS complexes (Fig. 5C–E). At the same time, I/R induced mitochondrial OXPHOS inhibition, evaluated by in vivo mitochondrial oxygen consumption analysis, was effectively improved after additional GCN5L1 knockdown (Fig. 5F). Consistent with the results in vivo, the effect of GCN5L1 on H/R induced mitochondrial dysfunction in TECs also showed the same tendency. As shown in Fig. 5G–M, GCN5L1 knockdown improved mitochondrial injury and elevated mtDNA copy number, mitochondrial protein expression and mitochondrial respiration capability in H/R induced TECs. On the whole, GCN5L1 deletion improves kidney dysfunction caused by acute kidney injury in vivo and in vitro.

### GCN5L1-mediated TFAM K76 acetylation participates in H/R-induced mitochondrial biogenesis impairment

**Fig. 6:**
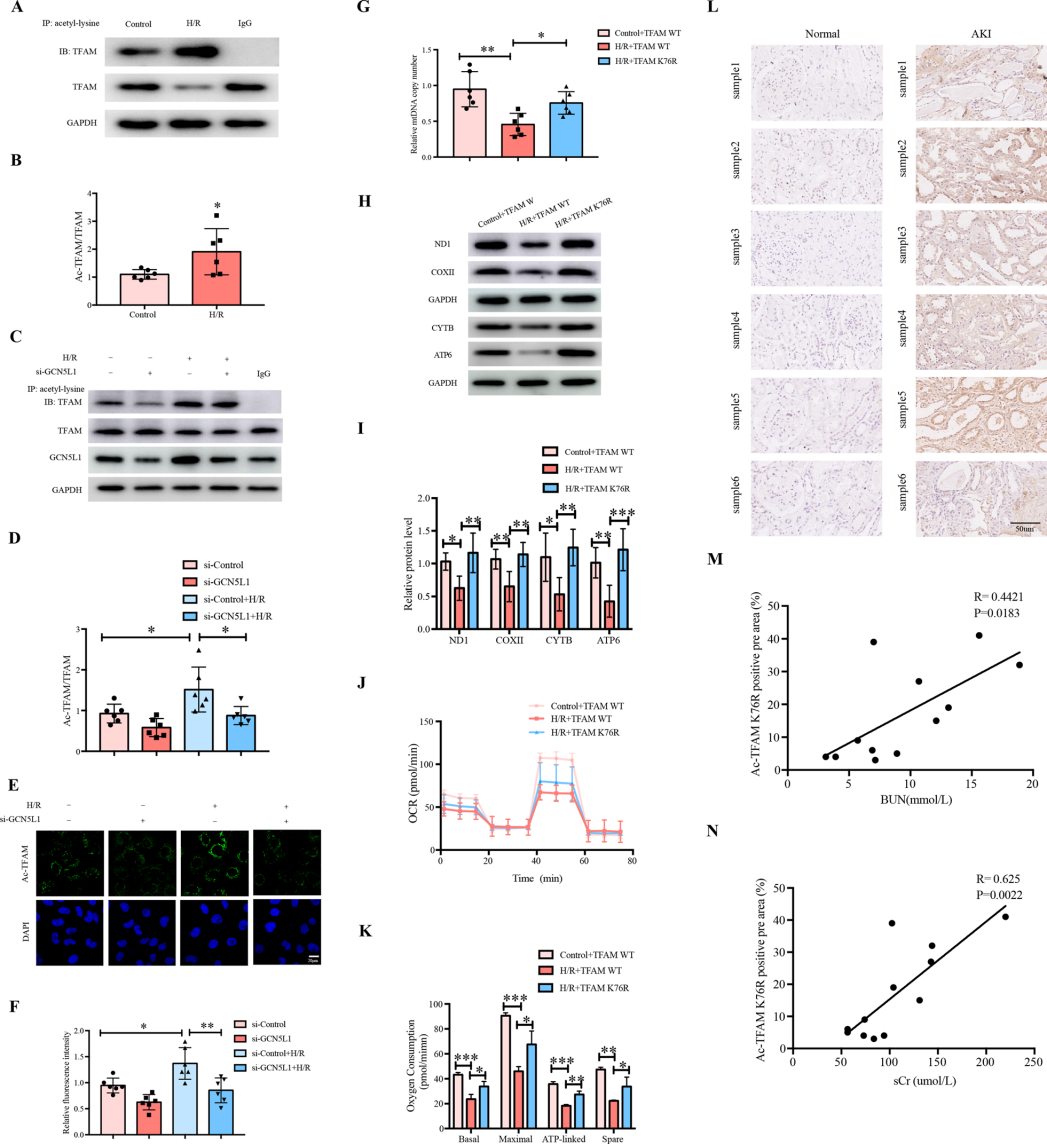
GCN5L1-mediated TFAM K76 acetylation participates in H/R-induced mitochondrial biogenesis impairment. **A**, **B** Acetylation level of TFAM was detected by immunoprecipitation in hypoxia-reoxygenation induced TECs. **C**, **D** Acetylation level of TFAM was detected by immunoprecipitation in GCN5L1 knockdown TECs under H/R treatment. **E**, **F** Acetylation level of TFAM was detected by Duolink proximity ligation assay in GCN5L1 knockdown TECs under H/R treatment. **G** mtDNA copy number in TECs transfected with TFAM K76 mutation plasmids under H/R treatment. **H**, **I** Protein levels of ND1, COXII, CYTB and ATP6 were detected by western blot in TECs transfected with TFAM K76 mutation plasmids under H/R treatment. **J**, **K** Bioenergetic profiles were measured by seahorse XF96 in TECs transfected with TFAM K76 mutation plasmids under H/R treatment. **L** Immunohistochemistry staining for acetylated TFAM K76 expression in kidney sections. **M** Positive correlation (Spearman r = 0.4421, P = 0.0183) between acetylated TFAM K76 IHC staining levels and BUN in all subjects. **N** Positive correlation (Spearman r = 0.625, P = 0.0022) between acetylated TFAM K76 IHC staining levels and serum creatinine in all subjects. *P < 0.05, **P < 0.01, and ***P < 0.001

The data presented above showed that GCN5L1 participated in AKI induced mitochondrial dysfunction and mediated TFAM K76 acetylation. Therefore, we suspect whether the effects of GCN5L1 on mitochondrial dysfunction are mediated by TFAM K76 acetylation. First, we found GCN5L1 knockdown effectively diminished H/R induced TFAM acetylation (Fig. 6A–F). Second, TFAM K76 mutant also exhibited protective effects on H/R induced mitochondrial injury similar to GCN5L1 knockdown, including increase of mitochondrial copy number (Fig. 6G), expression of mtDNA-encoded genes (Fig. 6H, I) and enhanced mitochondrial OXPHOS capability (Fig. 6J, K). More importantly, quantification of IHC staining demonstrated that the level of acetylated TFAM K76 was positively correlated with the serum creatinine (Spearman r = 0.625, P = 0.0022, n = 6) (Fig. 6M) and blood urea nitrogen (Spearman r = 0.4421, P = 0.0183, n = 6) (Fig. 6N) in AKI patients. Collectively, these data suggested that GCN5L1 mediated TFAM K76 acetylation, and accordingly TFAM mitochondria-importing inhibition, were involved in AKI induced mitochondrial injury.

## Discussion

Acetylation has emerged as the most prevalent PTM manner for mitochondrial function regulation [22] And evaluated about 35% mitochondrial proteins contain acetylation sites, and these proteins cover almost every aspect of mitochondrial biology, such as TCA cycle, oxidative phosphorylation, fatty acid oxidation, amino acid metabolism, carbohydrate metabolism, nucleotide metabolism and the urea cycle [23]. Compared with the well elucidation of mitochondrial deacetylase enzymes including SIRT3, SIRT4 and SIRT5, mitochondrial specific acetyltransferase was not identified and mitochondrial protein acetylation was supposed to be a nonenzymatic process, until the recent identification that GCN5L1 could interact with and acetylate several lines of mitochondrial proteins [24]. As a 15 kDa protein with sequence homologous to the nuclear acetyltransferase GCN5, GCN5L1 has a cellular distribution of both cytosol and mitochondria [25]. Cytoplasmic GCN5L1 mainly participates in the process of endosome–lysosome biogenesis, while mitochondria-localized GCN5L1 was evidenced to be capable of modulating multiple mitochondrial functions, such as fatty acid oxidation, gluconeogenesis and mitophagy [26–28]. A recent study also suggested that GCN5L1 might have impacts on mitochondrial biogenesis. In cultured mouse embryonic fibroblasts cells, Scott I and colleagues demonstrated that GCN5L1 deletion could induce the expression of peroxisome proliferator-activated receptor gamma coactivator 1-alpha (PGC-1α), and accordingly initiate mitochondrial biogenesis, while the concrete intermediate mechanism underlying the effects of GCN5L1 on PGC-1α remains to be determined [29]. Our results here also support the notion that GCN5L1 could negatively control mitochondrial biogenesis, as GCN5L1 deletion led to a significant elevation in mtDNA copies and cellular mitochondrial abundance, along with enhanced OXPHOS capability, while GCN5L1 overexpression exerted opposite effects. Furthermore, our present study demonstrated a direct mechanism accounting for the effects of GCN5L1 on mitochondrial biogenesis, as is GCN5L1 could acetylate TFAM at its K76 site, thereby inhibiting its binding to TOM70 and subsequent transporting into mitochondria, consequently reducing its mitochondrial accumulation (Fig. 4). These findings not only supplemented the current knowledge about the biology of GCN5L1, but also provided a novel modulating mechanism for intracellular transport of TFAM as well.

The acetylating modification of TFAM has been reported previously, while the detailed functional elucidation of this modulation manner is only beginning to emerge [30]. A previous study reported that TFAM could be acetylated at its K154 site within mitochondria, and this modulation could reduce its binding affinity to mtDNA, accordingly inhibiting its biological effects on mitochondrial genome [31]. In the present study, we demonstrated that GCN5L1-induced TFAM acetylation acted at another site, K76 site (Fig. 2). Furthermore, this modification occurred in the cytoplasm while not within mitochondria, and mainly affected the intracellular transport machinery of TFAM. Thus, it seemed that acetylating modification might be a crucial regulating manner for TFAM’s biology and acetylating at different TFAM molecular sites might exert different effects. Besides, the identification of GCN5L1 as a specific acetyltransferase for TFAM K76 site acetylation indicated that this modulation manner of the intracellular transport of TFAM is not a passive nonenzymatic process secondary to fluctuations of cellular acetyl-coenzyme A contents, but an enzyme-controlled active one. In addition, it should be mentioned that via using bioinformatics analysis, we screened a total of seven potential acetylating sites within TFAM. Besides K154 and K76, whether the remaining five sites could also be acetylated, along with their separate biological effects, are of interests deserving further exploration.

As a nuclear DNA-encoded protein, TFAM should be transported from the cytoplasm into mitochondria to execute its biological functions [32]. However, the intracellular transporting machinery for TFAM is of little known so far. A recent study reported that HSP70 might act as its chaperone facilitating its translocation to mitochondria [32]. We employed bioinformatics to search the potential interacting proteins of TFAM and screened a total of six candidate transporting proteins, including the previously reported HSP70, translocase of the outer membrane TOM70, TOM40, TOM20, and translocase of the inner membrane TIMM44 and TIMM17A. Our following co-immunoprecipitation studies revealed all these 6 proteins could bind to TFAM, thus outlining the intracellular transport machinery of TFAM (Fig. 4). Besides, the results showed that GCN5L1 obviously impaired the binding of TFAM to TOM70 without affecting its binding to HSP70, TOM40, TOM20, TIMM44 and TIMM17A, indicating TFAM K76 site acetylation might selectively affect the process of TFAM translocating through OMM. These results also coincided with the findings above that TFAM K76 site acetylation occurred in the cytosol and was mediated by cytoplasmic GCN5L1, although GCN5L1 is also known to have a mitochondrial distribution.

For cellular homeostasis maintenance, mitochondrial biogenesis must be finely tuned to match the different cellular energetic states. The process of mitochondrial biogenesis requires the expression of ample genes from both nuclear and mitochondrial genome, and tightly controlled by coordination between the two genomes as well [33]. Due to its nuclear DNA-origin property and multifaceted effects on mtDNA, TFAM was proposed as a major player in the adaptive process of mitochondrial biogenesis to environmental stresses. However, the concrete energetic-sensing mechanism of TFAM has not been fully elucidated. Current notion suggested that nuclear respiratory factor 1(Nrf1) and PGC-1α might act as the major regulators for TFAM expression and be involved in the energetic stresses-induced response of mitochondrial biogenesis [34]. Our results indicated that H/R-induced reduction of mitochondrial abundance was accompanied by a significant elevation of GCN5L1 expression and acetylated TFAM levels, and a concurrent decrease of mitochondrial TFAM contents, thereby suggesting that GCN5L1-mediated TFAM acetylation and TFAM trafficking modulation might be a novel sensing mechanism of mitochondrial biogenesis. Furthermore, such findings also coincided with the emerging role of GCN5L1 as the energetic sensor in modulating mitochondrial glucose and fatty acid oxidation, in response to nutrients challenges including both scarce and oversupply states of nutrients [16, 27].

The results that both in vitro hypoxia/reoxygenation and in vivo ischemia/reperfusion led to alterations of GCN5L1 expression indicated GCN5L1 might be one active player underlying the pathogenesis of ischemic AKI, and a potential intervening target as well. AKI is one of the most common kidney pathologies leading to high morbidity and mortality, with mitochondrial dysfunction serving as the major fundamental pathogenic event [35]. Experimental studies have revealed that five to ten minutes of ischemic attack could lead to significant inhibition of cellular respiration of TECs, with subsequent cellular ATP exhaustion and apoptotic or necrotic cell death [36]. On the other hand, rapid restoration of mitochondrial respiration, for example via promoting mitochondrial biogenesis, was proposed as the key intervening method for both preventing the onset of AKI and facilitating its recovery [37]. Currently, PGC-1α, a co-transcriptional regulation factor capable of inducing mitochondrial biogenesis by activating TFAM expression, is proposed as the one pharmacologic target, and several lines of agents aiming at PGC-1α including PPAR-γ agonist, β2-adrenergic receptor agonist and 5-HT2 agonists are under clinical evaluation or development for treating AKI [38]. Our results that kidney-specific knockdown of GCN5L1 was effective in ameliorating bilateral renal pedicle clamping induced kidney morphological and functional injuries, suggested that reducing GCN5L1 could serve as another intervening target, and agents specific acting on GCN5L1 deserve further exploration. Besides, the intermediate mechanisms responsible for I/R-induced GCN5L1 expression are also of significance for future intervening AKI.

## Conclusions

In conclusion, our present study demonstrated that GCN5L1 induced TFAM K76 site acetylation could modulate TFAM translocation into mitochondria, thereby affecting its effects on mitochondrial biogenesis, and that this mechanism participates in the energetic stresses-induced response of mitochondrial biogenesis.

## Supplementary Information

**Additional file 1: Table S1.** Antibodies. **Table S2.** Oligonucleotides. **Figure S1.** GCN5L1 negatively controls mitochondrial biogenesis.

## Data Availability

The data supporting the findings of this study could be obtained from the corresponding author.

## Abbreviations

GCN5L1: General control of amino acid synthesis 5 like-1
TFAM: Mitochondrial transcription factor A
AKI: Acute kidney injury
IRI: Ischemia reperfusion injury
I/R: Ischemia/reperfusion
H/R: Hypoxia-reoxygenation
TECs: Renal tubular epithelial cells
TOM70: Translocase of outer membrane 70
mtDNA: Mitochondrial DNA
UmtDNA: Urinary mitochondrial DNA
OXPHOS: Oxidative phosphorylation
sCr: Serum creatinine
BUN: Blood urea nitrogen
HE: Hematoxylin–eosin
HK-2: Human kidney cortex/proximal tubule cell
