# Supplementary material for: GCN5L1-mediated TFAM acetylation at K76 participates in mitochondrial biogenesis in acute kidney injury
Source: J Transl Med. 2022 Dec 6;20:571. doi: 10.1186/s12967-022-03782-0 (PMC9724393; doi:10.1186/s12967-022-03782-0)
Supplement: Supplementary file 1 — Additional file 1: Table S1. Antibodies. Table S2. Oligonucleotides. Figure S1. GCN5L1 negatively controls mitochondrial biogenesis. [file 12967_2022_3782_MOESM1_ESM.docx]

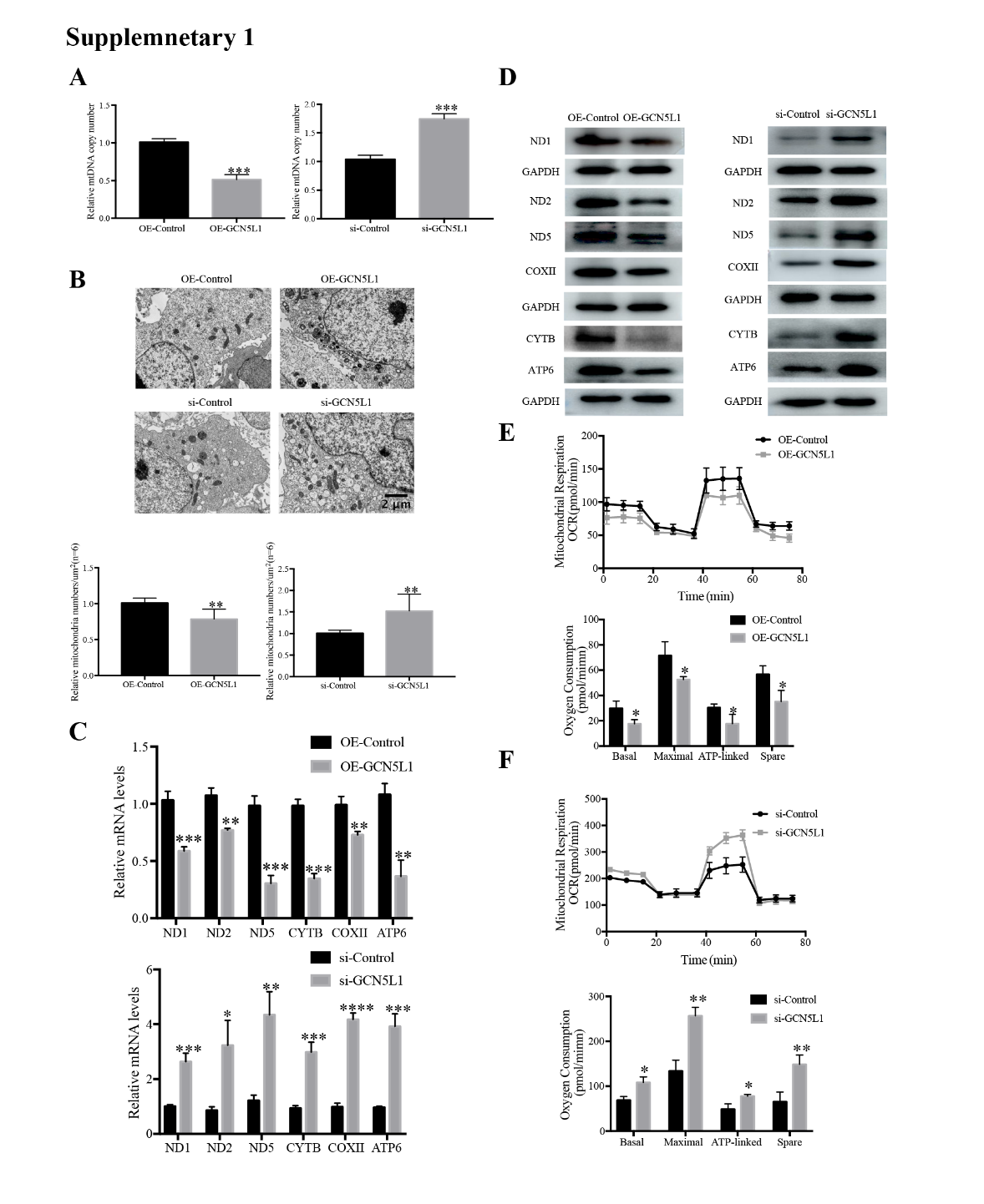


**Figure** **S1. GCN5L1 negatively controls mitochondrial biogenesis.**

**A** mtDNA copy number in TECs with GCN5L1 overexpression or knockdown.Two-tailed unpaired student’s t test was used, n=3, ***P<0.001. **B** Electron microscopy analysis for mitochondrial number and morphology in TECs with GCN5L1 overexpression or knockdown. Scale bars= 2 μm, two-tailed unpaired student’s t test was used, n=6, **P<0.01. **C** mRNA levels of ND1, ND2, ND5, CYTB, COXII and ATP6 were detected by qRT-PCR in TECs with GCN5L1 overexpression or knockdown. Two-tailed unpaired student’s t test was used, n=3, *P<0.05, **P<0.01, ***P<0.001. **D** Protein levels of ND1, ND2, ND5, COXII, CYTB and ATP6 were detected by western blotting in TECs with GCN5L1 overexpression or knockdown. **E, F** Bioenergetic profiles were detected by Seahorse XF96 in TECs after GCN5L1 was upregulated or downregulated. Mitochondrial OXPHOS was analyzed with basal respiration, maximal respiration, ATP production and spare respiratory capacity respectively. Two-tailed unpaired student’s t test was used, n=3, *P<0.05, **P<0.01.

| **Table S1: Antibodies** |  |  |
| --- | --- | --- |
| Mouse-β-actin (1:5000) | Proteintech | Proteintech Group Cat# 60008-1-Ig |
| Rabbit-GAPDH (1:5000) | Proteintech | Proteintech Group Cat# 10494-1-AP |
| Rabbit-VDAC (1:1000) | Proteintech | Proteintech Cat# 55259-1-AP |
| Mouse-GCN5L1 (1:500) | Santa Cruz | Santa Cruz Cat# sc-515444 |
| Rabbit-GCN5L1 (1:1000) | Proteintech | Proteintech Group Cat# 19687-1-AP |
| Rabbit-ND1 (1:1000) | Proteintech | Proteintech Group Cat# 19703-1-AP |
| Rabbit-ND2 (1:1000) | Proteintech | Proteintech Group Cat# 19704-1-AP |
| Rabbit-ND5 (1:1000) | Proteintech | Proteintech, Group Cat# 55410-1-AP |
| Rabbit-CYTB (1:1000) | Proteintech | Proteintech, Group Cat# 55090-1-AP |
| Rabbit-COXII (1:1000) | Proteintech | Proteintech, Group Cat# 55070-1-AP |
| Rabbit-ATP6 (1:1000) | Proteintech | Proteintech, Group Cat# 55313-1-AP |
| Rabbit-TFAM (1:5000) | Abcam | Abcam Cat # ab176558 |
| Mouse-TFAM (1:1000) | Abcam | Abcam Cat# ab119684 |
| Rabbit- Anti-acetyl Lysine (1:1000) | Cell Signaling Technology | Cell Signaling Technology Cat# 9441 |
| Mouse- Anti-acetyl Lysine (1:1000) | Abcam | Abcam Cat# ab22550 |
| Rabbit-HSP70 | Proteintech | Proteintech Group Cat# 10995-1-AP |
| Rabbit-TOM70 | Proteintech | Proteintech Group Cat# 14528-1-AP |
| Rabbit-TOM40 | Proteintech | Proteintech Group Cat# 18409-1-AP |
| Rabbit-TOM20 | Abcam | Abcam Cat# ab186735 |
| Rabbit-TIMM44 | Proteintech | Proteintech Group Cat# 13859-1-AP |
| Rabbit-TIMM17A | Abcam | Abcam Cat# ab192246 |
| Anti-mouse IgG, HRP-linked Antibody (1:5000) | Cell Signaling Technology | Cell Signaling Technology Cat# 7076 |
| Anti-rabbit IgG, HRP-linked Antibody (1:5000) | Cell Signaling Technology | Cell Signaling Technology Cat# 7074 |
| Alexa Fluor® 594 Conjugate (1:1000) | Cell Signaling Technology | Cell Signaling Technology Cat# 8890 |
| Alexa Fluor® 488 Conjugate (1:1000) | Cell Signaling Technology | Cell Signaling Technology Cat# 4408 |

| **Table S2: Oligonucleotides** | | | |
| --- | --- | --- | --- |
| GENENAME | Forward primers（5'-3'） | Reverse primers（5'-3'） | SOURCE |
| homo-β-actin | TGGCACCCAGCACAATGAA | CTAAGTCATAGTCCGCCTAGAAGCA | TAKARA |
| homo-GCN5L1 | ATTGGGGATGTGGAGAACTGG | TGAAGTAAGAGGGGTGGGTTTT | TAKARA |
| homo-TFAM | CAAGTTGTCCAAAGAAACCTGTAAG | GCCACTCCGCCCTATAAGC | BIOSUNE |
| homo-ND1 | CGAGCAGTAGCCCAAACAATC | GATGGCAGGAGTAATCAGAGGTG | BIOSUNE |
| homo-ND2 | ACCATCTTTGCAGGCACACT | GCTTCTGTGGAACGAGGGTT | BIOSUNE |
| homo-ND5 | TCAGTTGATGATACGCCCGA | TGGGGTGAGGCTTGGATTAG | BIOSUNE |
| homo-CYTB | CCCACCCCATCCAACATCTC | GCGTCTGGTGAGTAGTGCAT | BIOSUNE |
| homo-COXII | CATGAGCTGTCCCCACATTAG | CGGTCGTGTAGCGGTGAAA | BIOSUNE |
| homo-ATP6 | ACCACAAGGCACACCTACAC | TATTGCTAGGGTGGCGCTTC | BIOSUNE |
| Homo-G6PC (nDNA) | GGCTCTCAACTCCAGCATGT | AGGACGAGGGAGGCTACAAT | BIOSUNE |
| Homo-DLOOP2 (mtDNA) | CTGTCTTTGATTCCTGCCTCAT | GTGGCTGTGCAGACATTCAA | BIOSUNE |
| Mouse-COXI (mtDNA) | GCCCCAGATATAGCATTCCC | GTTCATCCT GTTCCTGCTCC | BIOSUNE |
| Mouse-β-actin (nDNA) | AGAGGGAAATCGTGCGTGAC | CAATAGTGATGACCTGGCCGT | BIOSUNE |
